# Synergistic impact of multi-frequency ultrasound on sea buckthorn juice fermentation: A holistic study on bioactive compounds, micromorphology, sensory quality, and computational evidence

**DOI:** 10.1016/j.ultsonch.2025.107571

**Published:** 2025-09-17

**Authors:** Sanabil Yaqoob, Aysha Imtiaz, Kanza Aziz Awan, Hiba Naveed, Ahmad Faraz, Remah Sobhy, Jian-Ya Qian, Akmal Nazir, Qing Shen

**Affiliations:** a Department of Food Science & Bioengineering, Zhejiang Gongshang University, Hangzhou 310018, China; b Panvascular Diseases Research Center, The Quzhou Affiliaed Hospital of Wenzhou Medical University, Quzhou People’s Hospital, Quzhou 324000, China; c Laboratory of Food, Nutrition and Clinical Research, Institute of Seafood, Zhejiang Gongshang University, Hangzhou 310012, China; d Department of Food and Nutritional Sciences, Faculty of Science and Technology, University of Central Punjab, Lahore, Pakistan; e School of Food Science and Engineering, Yangzhou University, Yangzhou, Jiangsu 225127, China; f Agricultural Product Processing and Storage Lab, School of Food and Biological Engineering, Jiangsu University, Zhenjiang, Jiangsu 212013, China; g Department of Biochemistry, Faculty of Agriculture, 13736, Moshtohor, Benha University, Egypt; h Department of Food Science, College of Agriculture and Veterinary Medicine, United Arab Emirates University, 15551, Al Ain, United Arab Emirates

**Keywords:** Sea buckthorn, Ultrasound-assisted fermentation, Lactic acid bacteria, Phenolic enrichment, Antioxidant activity, Functional beverages, Multi-frequency ultrasonication

## Abstract

This study investigates the combined effect of multi-frequency ultrasound-assisted fermentation on enhancing the functional and nutritional quality of sea buckthorn (*Hippophae rhamnoides* L.) juice. A defined tri-strain lactic acid bacteria (LAB) consortium; *Lactiplantibacillus plantarum*, *Lacticaseibacillus paracasei,* and *Lactobacillus helveticus* was employed to evaluate its impact on phytochemical enrichment, antioxidant potential, and sensory characteristics. The integrated bioprocess resulted in a substantial increase in total phenolic content (23.8-fold) and total flavonoid content (1.18-fold) compared to the unfermented control. Antioxidant activity showed significant enhancement with DPPH radical scavenging increasing from 88.63 % to 122.73 % and FRAP rising from 100.91 to 566.58 µmol/L. HPLC analysis revealed notable increases in quinic acid (1.29-fold), gallic acid (3.20-fold), and citric acid (7.34-fold), while FTIR, SEM, and XRD analyses confirmed microstructural alterations and improved bioactive accessibility. Sensory evaluation indicated elevated fruity and floral aroma intensities, with scores rising from 2.5 to 4.5. These findings demonstrate that multi-frequency ultrasound coupled with LAB fermentation is an effective, non-thermal approach for enhancing the bioactive, structural, and organoleptic attributes of sea buckthorn juice, supporting its potential as a next-generation functional beverage with broad nutraceutical applications.

## Graphical abstract

**Figure:**
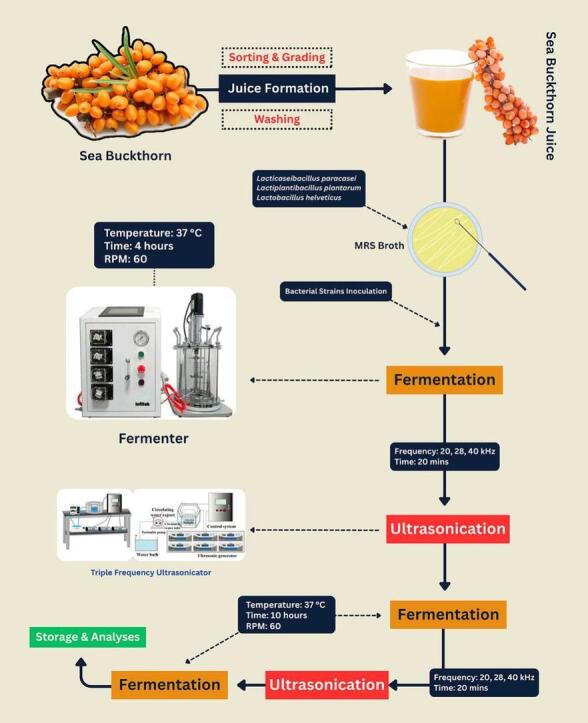

## 1 Introduction

Sea buckthorn (*Hippophae rhamnoides L*.), a member of the *Elaeagnaceae* family, is a hardy nitrogen-fixing shrub widely distributed across Europe and Asia. It has garnered considerable attention for its remarkable nutritional and therapeutic potential, deeply rooted in traditional medicine systems [1]. Due to its notable medicinal and industrial applications, sea buckthorn has been domesticated worldwide, including Europe, North America, China, Bhutan, Afghanistan, Mongolia, Nepal, Pakistan, and India. China is one of the leading producers and exporters of sea buckthorn, cultivating approximately 300,000 ha and harvesting from 740,000 ha. It yields around 8.5 million tons annually, generating about 1.43 billion USD in revenue. Mongolia follows, with 6,000 ha under cultivation and an additional 13,500 ha harvested from wild stocks, producing between 1,200 and 1,600 tons of sea buckthorn per year and generating roughly 5 million USD in revenue. [2]. Sea buckthorn is famous for its good antioxidant profile and diverse phytochemical components. These berries contain abundant hydrophilic antioxidant compounds such as phenolic compounds, flavonoids, and tocopherol, where their seeds contain a considerable quantity of lipophilic substances [3]. This distinctive combination of water- and fat-soluble antioxidants allow sea buckthorn to effectively combat a broad variety of reactive oxygen species (ROS) in the body, assisting a dynamic equilibrium between oxidant and antioxidant levels within a cell through different mechanisms to reduce the accumulation of free radicles and to mitigate the chances of oxidative stress [4]. Studies highlight the therapeutic implications of sea buckthorn, such as antioxidant mechanisms, cardioprotective behavior, anti-inflammatory properties, and dermo-protective effects, positioning sea buckthorn as an efficient ingredient to be used in the processing of functional foods and nutraceuticals [5].

Despite a well-documented bioactivity of sea buckthorn, its complete scientific and industrial exploitation remains limited due to several critical challenges. Among these is the insufficient characterization of phytochemical synergism, specifically the combined antioxidant and anti-inflammatory effects mediated by flavonoids, phenolic acids and polysaccharides [6,7]. Moreover, the lack of standardized extraction and analytical protocols further contributes to variability in reported bioactivities, hindering the reproducibility of the methods and translational applications. Current research lacks a detailed understanding of how sea buckthorns bioactive moieties work at the molecular level, including their specific interactions and metabolic pathways. Thus, bridging this gap through advanced phytochemical analysis and mechanistic studies could transform sea buckthorn from a traditional remedy into an evidence-based therapeutic option in managing conditions like oxidative stress, as well as in the formation of nutraceuticals. With the growing demand for nutrient-dense meals, multifrequency ultrasound (MFU) for fermentation is a novel method of producing functional beverages like sea buckthorn juice. This procedure elevates the concentration of bioactive constituents in the juice and resolves different manufacturing and preservation challenges, enabling consumers to indulge in these delicious and nourishing beverages.

Recent advances in non-thermal processing, particularly MFU, offer a promising route to enhance the bioavailability of phytochemicals. MFU generates intense acoustic cavitation, resulting in localized high-temperature and high-pressure micro zones that improve cell wall permeability, enzymatic activity, and mass transfer without compromising thermolabile components [8]. When coupled with lactic acid bacteria (LAB) fermentation, ultrasound-assisted processing can synergistically enhance the release, transformation, and stability of bioactive compounds. Integration of ultrasound-assisted microbial fermentation has been demonstrated to markedly enhance the extraction and bioavailability of phenolic compounds, elevate antioxidant capacity and improve the organoleptic properties of plant-based commodities [9,10]. Practical applications of ultrasound-assisted fermentation have demonstrated promising results in enhancing the functional quality of sea buckthorn juice. For instance, dual-frequency ultrasonication (20/40 kHz) has been shown to increase flavonoid yields by 47 % while simultaneously reducing fermentation time by 35 % [11]. Additionally, the use of sweep-frequency ultrasound in sea buckthorn fermentations resulted in a 2.3-fold improvement in anthocyanin retention and a significant elevation in the production of moracin derivatives, particularly morusin, a potent AMPK activator with established anti-diabetic properties, reaching concentrations up to 1.8 mg/L [12].

The primary aim of this study is to investigate the effects of multi-frequency ultrasound (MFU) on the fermentation process of sea buckthorn juice using *Lactobacillus plantarum*. Specifically, we evaluated changes in bioactive compound profiles, micromorphological transformations, and sensory quality. Mechanistic insights were supported via *in-silico* molecular docking and modeling techniques. The ultimate objective is to assess the feasibility of developing a functionally enhanced fermented beverage with improved nutritional and sensory properties.

## 2 Materials and methods

### 2.1 Procurement of raw material

Fresh sea buckthorn (*Hippophae rhamnoides* L.) berries were procured from local markets in Hangzhou, Zhejiang, China. Fully mature fruits were manually selected based on uniform size and absence of physical defects. The berries were thoroughly washed, peeled, and stored at − 18 °C until further processing. Juice was extracted using a laboratory-scale blending machine before subsequent experimental treatments. *Lactiplantibacillus plantarum*, *Lacticaseibacillus paracasei,* and *Lactobacillus helveticus*, which were bought from SynBio Tech, Beijing, China, were propagated on MRS medium at 37 °C and stored at 4 °C. All the employed chemicals or solvents were of analytical grade.

### 2.2 Multifrequency ultrasonication-assisted fermentation conditions

**Fig. 1:**
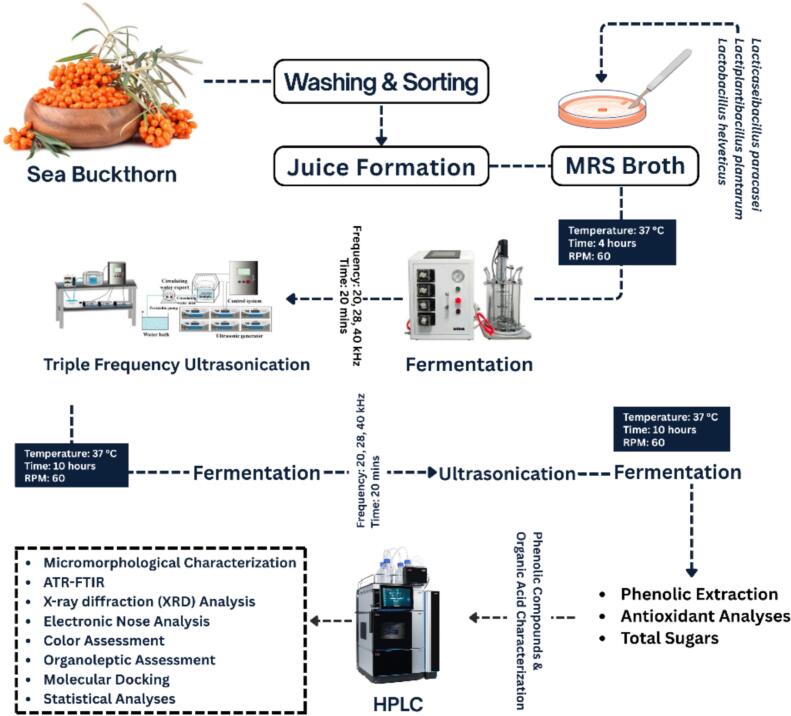
Flowchart illustrates the methodology followed for the ultrasound assisted fermentation and analytical evaluation of sea buckthorn juice using selected bacterial strains.

Sea buckthorn juice was subjected to multifrequency ultrasound-assisted fermentation based on previously optimized parameters, as expressed in Fig. 1. Three probiotic strains, *Lactiplantibacillus plantarum*, *Lacticaseibacillus paracasei,* and *Lactobacillus helveticus,* were individually cultured in MRS broth within an HZQ-F160 incubator until reaching the logarithmic growth phase (10^7^–10^8^ CFU/mL). A 2 % (v/w) bacterial inoculum was aseptically introduced into the juice which was prior heated at 95 °C for 1 min under continuous stirring, followed by primary fermentation at 37 °C with orbital shaking at 60 rpm for 4 h. Subsequently, ultrasonication was performed using a tri-frequency ultrasonic bath (20/28/40 kHz; 50 W/L) in pulsed mode (10 s on/10 s off) for a total of 20 min. The temperature was maintained at 25 ± 2 °C using a water circulation system. Following ultrasonication, a secondary fermentation phase was carried out for an additional 10 h, completing a 24-h cycle of alternating fermentation and ultrasound treatment [14].

### 2.3 Phenolic extraction

Free phenolic compounds were extracted following the method described by Dou, Chen, Fu and Liu [15] with minor modifications. Briefly, 1.0 g of the sample was mixed with 20 mL of 80 % (v/v) methanol and subjected to ultrasonication for 30 min. The mixture was then centrifuged at 4000 × g for 10 min. The resulting supernatant was collected, lyophilized, and stored for subsequent analyses as the undigested phenolic fraction.

#### 2.3.1 Total phenolic contents (TPC)

The TPC was quantified following the Folin-Ciocalteu method as mentioned by Chen, Chen, Xiao and Fu [16]. 200 μL of sample extract was combined with 1 mL of Folin-Ciocalteu reagent, 3 mL of 20 % (w/v) sodium carbonate solution, and 12 mL of distilled water to form the reaction mixture. The solution was vortexed thoroughly and incubated at 70 °C for 10 min in a water bath. Absorbance measurements were performed at 765 nm using a SpectraMax i3 microplate reader (Molecular Devices, San Jose, CA, USA). Quantification was achieved using a gallic acid calibration curve (0–500 μg/mL) with results expressed as milligrams of gallic acid equivalents per milliliter of sample (mg GAE/mL).

#### 2.3.2 Total flavonoid contents (TFC)

The TFC was measured from the sample using the procedures outlined by Chen, Chen, Xiao and Fu [16]. A mixture of 100 μL of distilled water, along with 10 μL of 5 % sodium nitrite solution and 25 μL of the sample, was allowed to stand for 5 min. Following that, 15 μL of 10 % aluminum chloride and 50 μL of sodium hydroxide and distilled water were added. Utilizing the SpectraMax i3 spectrophotometer from Molecular Devices, Silicon Valley, CA, USA, absorbance measurements were performed at 510 nm. Quercetin was used as a reference to determine TFC, and the results were reported as milligrams of rutin equivalents (mg RE) per milliliter of sample.

#### 2.3.3 Total flavonol content (TflavC)

The total flavonol content of sea buckthorn juice was ascertained using the method of Yaqoob, Imtiaz, Khalifa, Maqsood, Ullah, Shahat, Al-Asmari, Murtaza, Qian and Ma [14] with slight modifications. Briefly, 3 mL of sodium acetate (CH_3_COONa), 2 mL of 10 % aluminum chloride (AlCl_3_), and 2 mL of the sample extract were mixed and incubated at 20 °C for 150 min. The absorbance was measured at 440 nm using a SpectraMax i3 spectrophotometer. Flavonol content was quantified using a quercetin calibration curve (concentration range: 31.25–500  µg/mL), and results were expressed as milligrams of quercetin equivalents (mg QE/mL) of sample.

#### 2.3.4 Determination of DPPH scavenging activity

The 2,2-diphenyl-1-picrylhydrazyl free radical scavenging activity of the sample was assessed using the technique outlined by Ramirez, Zambrano, Sepúlveda, Kennelly and Simirgiotis [17]. 4.2 mL of a 0.1 mM DPPH solution prepared in methanol (MeOH), 120 µL of the sample was added. The combination was then placed in the dark for 1.5 h at room temperature. To find the absorbance at 517 nm, a UV–Vis spectrophotometer was employed. A sample without DPPH was employed as a blank. To calculate the radical scavenging activity, the following formula was used:

(1) $$DPPH\;scavenging\;activity\;\left(\%\right)=\left(\frac{A_{o}-A_{s}}{A_{0}}\right)\times 100$$

where, $A_{o}$ is the absorbance of the control, and $A_{s}$ is the absorbance of the sample.

#### 2.3.5 Determination of ferric reducing antioxidant power (FRAP)

The FRAP of samples was evaluated following Hsieh and Rajashekaraiah [18]. 25 mL of acetate buffer (0.3 M, pH 3.6), 2.5 mL of 10 mm TPTZ solution in 40 mm HCl, and 2.5 mL of 20 mM FeCl_3_·6H_2_O were combined to create the FRAP reagent. 1.0 mL of the sample and 0.3 mL of distilled water were combined with 0.3 mL of FRAP reagent to create the test. For 30 min, the finished reaction mixture was submerged in a water bath at 37 °C. A UV–Vis spectrophotometer was then used to measure absorbance at 595 nm. The results were then represented as milligrams of Trolox equivalents per milliliter of sample (mg TE/mL).

#### 2.3.6 Evaluation of proanthocyanidin content (PAC)

The proanthocyanidin concentration of sea buckthorn juice was determined following the method described by Esquivel-Alvarado, Muñoz-Arrieta, Alfaro-Viquez, Madrigal-Carballo, Krueger and Reed [19] with slight modifications. Briefly, 1  mL of the sample was mixed with 3  mL of concentrated hydrochloric acid and 6  mL of vanillin reagent. The mixture was incubated at 25 °C for 15 min. Absorbance was then measured at 500  nm using a SpectraMax i3 spectrophotometer. Proanthocyanidin content was quantified using a catechin standard curve and expressed as milligrams of catechin equivalents per milliliter (mg CE/mL) of sample.

#### 2.3.7 Determination of H_2_O_2_ scavenging activity

The hydrogen peroxide (H_2_O_2_) scavenging activity of sea buckthorn juice was assessed following the method described by Özyürek, Bektaşoğlu, Güçlü, Güngör and Apak [20] with slight modifications. Briefly, 2.0  mL of the sample was mixed with 2.0  mL of a 40  mM hydrogen peroxide solution prepared in phosphate buffer (pH 7.4). The mixture was incubated at room temperature for 10 min. After incubation, the absorbance was measured at 230  nm using a UV–Vis spectrophotometer. A greater decrease in absorbance indicated a higher H_2_O_2_ scavenging capacity of the sample extract.

#### 2.3.8 CuCl_2_ reducing power ability

The CuCl_2_ activity of sea buckthorn samples was evaluated using the methodology depicted by Murtaza, Yaqoob, Mubeen, Sameen, Murtaza, Rehman, Alsulami, Korma, Khalifa and Ma [21], with slight modifications. Shortly, a mixture was prepared with 250 μL of CuCl_2_ (0.01 M), 250 μL of ammonium acetate (1 M), 250 μL of neocuproine (7.5 M), and varying quantities of the treatments. The solution was left to stand at room temperature for 30 min, because of which the absorbance was observed at 450 nm.

#### 2.3.9 Reducing power

The reducing power of sea buckthorn treatments was evaluated using a modified method based on Natić, Dabić, Papetti, Akšić, Ognjanov, Ljubojević and Tešić [22]. Briefly, 1  mL of the sample was mixed with 50  μL of hydrochloric acid (HCl), 400  μL of ferric chloride (FeCl_3_), 400  μL of potassium ferricyanide (K_3_[Fe (CN)_6_]), and 700  μL of distilled water. The resulting mixture was incubated in the dark at 37 °C for 30 min. After incubation, the absorbance was measured at 720  nm using a SpectraMax i3 spectrophotometer. The reducing power was quantified using a standard curve of ascorbic acid and expressed as millimolar concentrations of ascorbic acid equivalents (mM AAE).

### 2.4 Total sugars

The total sugar content of sea buckthorn samples was determined using the phenol–sulfuric acid method as described by Lam, Dinh and Dang-Bao [23]. Briefly, 100  μL of the dry ethanolic extract was mixed with 0.5  mL of distilled water and 0.5  mL of 5 % phenol solution. Subsequently, 2.5  mL of concentrated sulfuric acid (H_2_SO_4_) was carefully added to the mixture. After thorough mixing, the solution was allowed to stand at room temperature for 30 min. The absorbance was then measured at 490  nm using a UV–Vis spectrophotometer. Quantification was performed using a standard calibration curve of pure D-glucose, and results were expressed as milligrams of glucose equivalents per milliliter of sample (mg GE/mL).

### 2.5 Characterization of phenolic compounds in sea buckthorn juice by HPLC

Phenolic compounds were evaluated using an Agilent 1260 Infinity II HPLC-UV system, following the method of Dou, Chen, Fu and Liu [15]. Separation was achieved using a C18 column (Agilent Zorbax SB, 4.6 × 250  mm, 5  µm particle size). The mobile phases consisted of solvent A (0.1 % acetic acid in water) and solvent B (100 % acetonitrile) which were applied in a linear gradient program as follows: 5–10 % B for 0–10 min; 10–15 min, 10–20 % B; 15–25 min, 20–38 % B; 25–30 min, 38–40 % B; 30–31 min, 40–100 % B; 31–35 min, 100 % B; 35–36 min, 100–5 % B and 36–50 min, 5 % B. A column temperature of 30 °C was established, and the flow rate was kept constant at 0.8 mL/min. General phenolics were detected at 250 nm, while flavonoids at 284 nm, and anthocyanins at 520 nm. Calibration curves based on peak areas were used to compute concentrations, and UV spectra and retention durations were compared with those of validated standards for identification and quantification.

### 2.6 Characterization of organic acids in sea buckthorn juice using HPLC

The quantification of organic acids (tartaric, malonic, oxalic, fumaric, succinic, lactic, and citric acids) was performed using high-performance liquid chromatography (HPLC) following El-Sohaimy, Shehata, Mathur, Darwish, Abd El-Aziz, Gauba and Upadhyay [24]. The analysis employed an Agilent 1100 series HPLC system equipped with a degasser, quaternary pump, autosampler (20 μL injection volume), and a reverse-phase C18 column (150 × 4.6 mm, 3 μm particle size) maintained at 25 °C with detection achieved using a diode array detector. Separation was carried out under isocratic conditions using 0.01 M KH_2_PO_4_ buffer (pH 2.6, adjusted with o-phosphoric acid) as the mobile phase at a flow rate of 0.5 mL/min with detection wavelengths set at 210 nm. Method validation demonstrated excellent precision through repeatability (10 consecutive injections) and intermediate precision (18 injections conducted over multiple days spanning six months) while accuracy was confirmed via recovery tests at two concentration levels. The method showed good sensitivity with detection and quantification limits corresponding to signal-to-noise ratios of 3:1 and 10:1, respectively. Positive identification of all analytes was achieved through retention time matching, UV–Vis spectral comparison, and standard addition experiments.

### 2.7 Micromorphological characterization of sea buckthorn samples

The micromorphological properties of the sea buckthorn samples were analyzed using a scanning electron microscope (Phenom™, Thermo Fisher Scientific) at a magnification of × 5000, following the protocol by Yaqoob, Liu, Liu, Zheng, Awan, Cai and Liu [25]. Freeze-dried samples were mounted onto metal stubs using double-sided conductive carbon tape. A thin layer of gold was applied via sputter coating to enhance conductivity. Imaging was performed under vacuum conditions at an accelerating voltage of 5  kV.

### 2.8 Attenuated total reflectance-Fourier transform infrared spectroscopy (ATR-FTIR)

Attenuated total reflectance-Fourier transform infrared (ATR-FTIR) spectroscopy was conducted to investigate the functional groups present in sea buckthorn juice using a Nicolet iS50 FTIR spectrometer (Thermo Scientific, Waltham, MA, USA) equipped with an ATR accessory. Spectral data were collected in the range of 4000–700 cm^−1^, following the method described by Rehman, Khalifa, Rasheed, Iqbal, Shoaib, Wang, Zhao, Liang, Zhong and Sun [26]. Secondary structural elements were specifically analyzed within the amide I region (1600–1700 cm^−1^). To extract compositional and structural information, spectral deconvolution and curve fitting were performed using PeakFit™ Version 4.0 and OMNIC™ software (Version 32).

### 2.9 X-ray diffraction (XRD) analysis

The crystalline structure of the sample was characterized using X-ray diffraction on a Rigaku AXS SmartLab diffractometer, following the method of Xie, Chen, Jiang, Zhou, Guo, Zeng and Zhang [27]. The analysis was conducted at 40 kV and 40 mA using Cu Kα radiation (λ = 0.15405 nm). The diffraction patterns were recorded over a 2θ range of 3° to 50° with a step size of 0.02° and a scanning speed of 10° per min. The relative crystalline was determined by calculating the ratio of the integrated area under the crystalline peaks to the total diffraction area, offering insights into the structural order within the samples.

### 2.10 Electronic nose analysis

Volatile compound analysis was performed using a PEN3 electronic nose (Airsense Analytics GmbH, Germany) following an adapted Yu, Huang, Wang, Ren, Zhang and Wang [28] protocol. Samples (10 mL) were equilibrated in 50 mL sealed vials before analysis. Operating parameters included: 120 s measurement time, 150 s sensor purge, 5 s pre-incubation, with carrier and injection gas flows of 400 and 200 mL/min, respectively. The MOS sensor array enabled VOC fingerprinting and discrimination.

### 2.11 Color assessment

Color parameters of sea buckthorn samples were measured using a HunterLab ColorQuest XE Spectrophotometer (Hunter Associates Laboratory, Virginia, USA) based on the CIE color space system, following the method of Boateng, Zhang, Li, Saalia and Yang [29]. The evaluated parameters included lightness (L*), redness (a*), and yellowness (b*). Additionally, the total color difference (ΔE) was calculated to assess the overall chromatic variation among treatments.

### 2.12 Organoleptic assessment

The sensorial characteristics of the sea buckthorn juice were evaluated using quantitative descriptive analysis (QDA), following the method of Yu, Huang, Wang, Ren, Zhang and Wang [28]. A total of 50 untrained panelists (25 males and 25 females), aged 18–30 years, were recruited from Zhejiang Gongshang University. All participants provided written informed consent before the evaluation. To minimize bias, samples were blind-coded using randomized three-digit identifiers and presented in a counterbalanced order. Panelists assessed the juice samples based on five defined sensory attributes: fruity (e.g., banana, peach, mulberry), floral (e.g., rose, violet), mellow (ethanol-like), estery (cheese-like), and delicate (e.g., grass, green bean). Attribute intensities were rated using a standardized 6-point Likert-type scale (0 = absent to 5 = pronounced). Sensory data were subjected to multivariate statistical analysis to identify dominant descriptors and generate sample-specific sensory profiles.

### 2.13 Molecular docking

The molecular docking technique was conducted *via* the available version of Discovery Studio software. The molecular interactions were performed between the structure of bacterial peptidoglycan (PDB ID: 2MTZ) as macromolecule receptor and Quinic acid (QA; PubChem CID: 37439) as ligand, which were retrieved protein data bank and PubChem databases (https://www.rcsb.org; https://pubchem.ncbi.nlm.nih.gov). Both structures were then optimized by combining fractional charges, and energy was minimized using Protonate-3D and MMFF94X force fields, and H_2_O was removed, structure refining, energy minimization, and 3D protonation [30]. The process was repeated for the composites, and thereafter, 4–5 appropriate docked postures were created, which were visualized and analyzed for their hydrophobicity, electrostatic potential, and H-bonds.

### 2.14 Data analysis

Statistical analyses were performed using IBM SPSS Statistics (Version 22; Armonk, NY, USA) and OriginPro 2022 (OriginLab Corp., Northampton, MA, USA). Data are presented as mean ± standard deviation (SD) of triplicate measurements. A two-way analysis of variance (ANOVA) was applied to determine the significance of the effects of fermentation time with different strains on the measured parameters as tabulated in Table 1. The post-hoc analysis using Tukey's test was performed following any significant ANOVA result (p < 0.05). Furthermore, multivariate pattern recognition was conducted using principal component analysis (PCA) in SIMCA-P + software (Version 14.1; Umetrics, Umeå, Sweden) to assess overall inter-group variability.

**Table 1:** Description of sea buckthorn juice treatments (SBo–SB7) based on ultrasound application and LAB strain combinations.

**Treatments**	**Strains**
SB_o_	Unfermented control
SB_1_	Ultrasound-treated sample
SB_2_	Ultrasound-assisted fermentation by *Lactiplantibacillus plantarum*
SB_3_	Ultrasound-assisted fermentation by *Lactiplantibacillus casei*
SB_4_	Ultrasound-assisted fermentation by *Lactobacillus helveticus*
SB_5_	Ultrasound-assisted fermentation by *Lactiplantibacillus plantarum* and *Lactiplantibacillus casei*
SB_6_	Ultrasound-assisted fermentation by *Lactiplantibacillus plantarum* and *Lactobacillus helveticus*
SB_7_	Ultrasound-assisted fermentation by *Lactiplantibacillus casei* and *Lactobacillus helveticus*

## 3 Results and discussion

### 3.1 Antioxidant assay of sea buckthorn

**Fig. 2:**
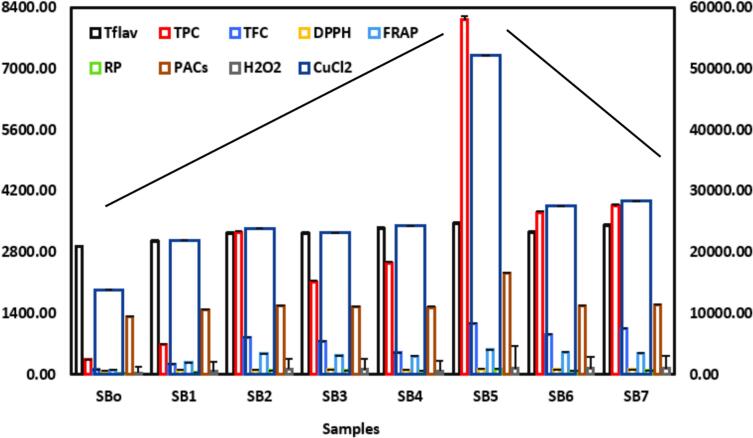
Antioxidant parameters of sea buckthorn juice (SBo–SB7), including TPC, TFC, TflavC, PAC, DPPH, FRAP, H_2_O_2_ scavenging, CuCl_2_ reduction and reducing power. SB5 showed the highest antioxidant activity across most indices. Means ± SD were represented, n = 3 replicates.

The antioxidant potential of sea buckthorn juice samples (SB_o_–SB_7_) demonstrated significant enhancement across multiple biochemical parameters, as illustrated in Fig. 2. Total flavonoid content (TFC) increased from 2922.27  µg/mL in the unfermented control (SB_o_) to 3455.27  µg/mL in SB_5_. A pronounced rise in TPC was observed in the same sample, reaching 8109.74  µg/mL compared to 340.38  µg/mL in SB_o_, representing a 23.8-fold increase. Similarly, TFC peaked at 1158.48  µg/mL in SB_5_, showing a tenfold increase over the control. Flavonol content exhibited the greatest increase, exceeding 75,000  µg QE/mL in SB5, whereas the control sample (SB_o_) contained less than 15,000  µg QE/mL. In terms of antioxidant assays, DPPH radical scavenging activity increased markedly from 88.63 % in the control to 122.73 % in SB5. FRAP also exhibited a notable rise from 100.91  µmol/L to 566.58  µmol/L, while reducing power (RP) improved from 23.33  µmol to 131.45  µmol. The Cu^2+^ reducing capacity (CuCl_2_ assay) spiked significantly in SB_5_, reaching 52,133.89  µmol compared to 13,890.56  µmol in SB_o_. Proanthocyanidin content (PAC) increased from 1314.50 to 2317.17  µg/mL, and H_2_O_2_ scavenging activity rose from 35.67 % to 141.33 % in SB_5_, the observed differences were statistically substantial at *p* < 0.05.

Overall, SB_5_ emerged as the most effective treatment, exhibiting the highest antioxidant performance across all evaluated indices. This enhancement is attributed to the enhanced effect of multi-frequency ultrasound and co-culture LAB fermentation, which facilitated improved release, transformation, and structural modification of bioactive compounds. The results confirm that ultrasound-assisted fermentation is a promising non-thermal strategy to maximize the antioxidant efficacy of sea buckthorn juice.

The present findings are consistent with those reported by Devendran, Abirami and Priyanga [31], who observed high levels of TPC and TFC in sea buckthorn fruits grown in the Kyrgyz Republic, accompanied by strong antioxidant activity as reflected in DPPH, FRAP, and CuCl_2_ assays. Similarly, [32] investigated the impact of drying techniques on sea buckthorn berries and concluded that freeze-drying effectively preserved higher levels of TPC and TFC, along with superior antioxidant capacity, highlighting the importance of post-harvest processing in maintaining bioactive integrity. Luntraru, Apostol, Oprea, Neagu, Popescu, Tomescu, Mulțescu, Susman and Gaceu [33], demonstrated that sea buckthorn by-products showed strong interaction between TPC and CUCL_2_ and FRAP, while TFC and Tflav also considerably influenced antioxidant activity, confirming their functional value in the antioxidative system. Wu, Xia, Huang, Tian, Ye and Wang [34], observed that centrifugation and transmembrane treatments influenced the polyphenols quantification and antioxidant readings in sea buckthorn juice, particularly affecting the values of DPPH, TPC, ABTS and FRAP, thus highlighting the sensitivity of antioxidant evaluations to sample preparation methods. However, the changes in results across studies suggest that antioxidant expression is highly dependent on the following parameters: extraction conditions, processing, and fruit origin. In short, the antioxidant profiling results revealed significant improvements in the bioactive composition and radical scavenging activity of sea buckthorn following processing. The marked increases in TPC, TFC, DPPH, FRAP, PACs, and H_2_O_2_, showed improvement in antioxidant efficacy. The co-culture bacteria *L. plantarum*, *L. helveticus,* and *L. paracasei*, which have been used to facilitate the release and modifications of antioxidant compounds during fermentation. These strains' combined enzymes and microbes enhance sea buckthorn phenolic bioavailability and functional quality via structural breakdown. To promote the potential industrial application of ultrasound-assisted fermentation for the development of functional juice, it is essential to examine the current outcomes in the context of established commercial standards. The improvement of antioxidant assay, particularly the enhanced phenolic content and radical scavenging activity is associated with the growing industry demand for functional beverages with proven health benefits.

### 3.2 Total sugars of sea buckthorn

**Fig. 3:**
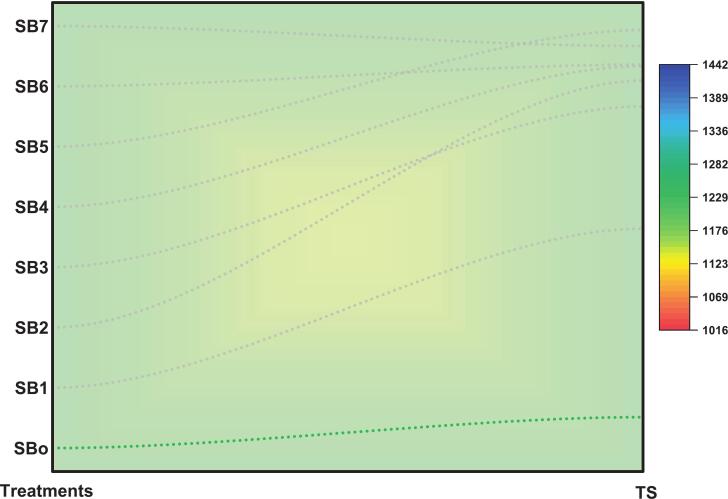
Total sugar content (mg GE/mL) of sea buckthorn juice samples (SBo–SB7) after ultrasound-assisted fermentation. SB5 exhibited the highest sugar concentration, indicating enhanced saccharification and carbohydrate release through combined microbial and ultrasonic treatment, n = 3 replicates.

The total sugar content of sea buckthorn samples showed a marked increase following ultrasound-assisted fermentation, as illustrated in Fig. 3**.** The unfermented control (SB_o_) recorded 1232.18  mg GE/mL, while treated samples (SB_1_–SB_7_) exhibited significantly higher values. Notably, SB_1_ showed an immediate increase to 2035.62  mg GE/mL, indicating a strong effect of ultrasound treatment alone. A progressive rise was observed across treatments, peaking at SB_5_ at 2882.64  mg GE/mL, where a 134 % increase over the control. Although SB_6_ (2735.18  mg GE/mL) and SB_7_ (2814.38  mg GE/mL) showed slightly lower values than SB_5_, they remained elevated. These results suggest that the combined ultrasound and fermentation treatments enhanced sugar release through improved polysaccharide breakdown and saccharification the noted differences were statistically significant at *p* < 0.05.

The observed increase in total sugar content among sea buckthorn treatments is consistent with previous findings on similar processing interventions. El-Sohaimy, Shehata, Mathur, Darwish, Abd El-Aziz, Gauba and Upadhyay [24], reported that fermentation of sea buckthorn juice with *Lactobacillus plantarum* enhanced sugar release from 1120.5  mg GE/mL in control to 1876.3  mg GE/mL post-fermentation, attributed to microbial enzymatic hydrolysis of complex carbohydrates into simpler, more bioavailable forms. This aligns with the present study, where both ultrasound and fermentation treatments significantly elevated sugar levels. Additionally, Wen, Huang, Li, Jiang, Shao, Zhang and Shan [35], demonstrated that ultrasound combined with enzymatic extraction improved sugar yields from sea buckthorn polysaccharides, with concentrations rising from 1024.7 to 1984.2  mg GE/mL, primarily due to enhanced cell wall disruption and carbohydrate release. These findings support the role of ultrasound-assisted fermentation in promoting saccharification, mediated by the enzymatic activities of *L. plantarum*, *L. helveticus* and *L. paracasei*. The enhanced action of these LAB strains, in combination with ultrasonic cavitation, appears to facilitate efficient breakdown of polysaccharides, increasing free sugar availability. Collectively, the consistency across studies highlights the potential of integrating probiotic fermentation with ultrasound processing to enhance the nutritional and functional profile of sea buckthorns and related plant-based matrices.

### 3.3 Determination of phenolics by utilizing HPLC of sea buckthorn

**Fig. 4:**
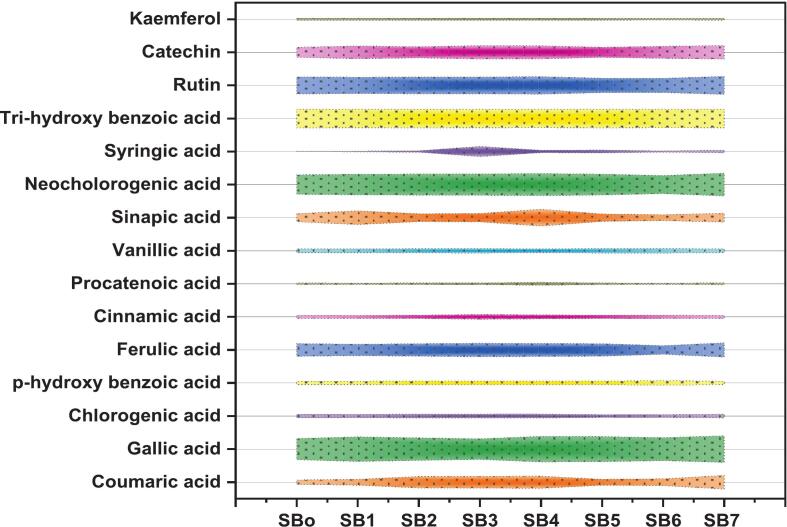
HPLC-based phenolic compound profiles of sea buckthorn juice (SBo–SB7), showing treatment-induced variations in gallic, ferulic, coumaric, catechin, rutin and other key phytochemicals. The Quinic acid values are 15909.75, 17276.28, 20591.46, 16399.90, 18207.74, 20432.66, 19081.11, and 17636.98 µg/mL in SBo, SB1-7, respectively, n = 3 replicates.

Ultrasound-assisted fermentation significantly altered the phenolic and organic acid profiles of sea buckthorn samples (SB_o_–SB_7_), as shown in Fig. 4. Quinic acid was the dominant organic acid, increasing from 15,909.75  µg/mL in the unfermented control (SB_o_) to 20,591.46  µg/mL in SB_2_, with SB_5_ (20,432.66  µg/mL) also showing elevated levels, indicating improved bioavailability or stimulated biosynthetic activity. Among phenolic acids, gallic acid exhibited a consistent increase, peaking at 66.23  µg/mL in SB_7_, while ferulic acid reached its maximum (35.68  µg/mL) in the same treatment. Coumaric acid showed a substantial threefold rise, from 11.10  µg/mL in the control to 33.47  µg/mL in SB_7_, suggesting effective release from bound forms. These substantial improvements in phenolic acid concentrations can be attributed to microbial enzymatic activities particularly *tannase*, which hydrolyze tannins into simpler phenolic substances like gallic and ellagic acids. Moreover, the enzymes like *feruloyl esterase* and *cinnamoyl esterase* produced by *Lactobacillus* species may contribute to the liberation of ferulic and coumaric acids from plant cell wall complexes. Flavonoid profiling revealed similar enhancements: catechin concentration rose to 34.17  µg/mL and rutin to 44.76  µg/mL in SB_7_. Neochlorogenic acid displayed a gradual increase across treatments (48.64 to 56.85  µg/mL), while kaempferol remained unchanged (4.62–5.26  µg/mL). Chlorogenic acid exhibited moderate fluctuations (7.44–10.23  µg/mL), and sinapic acid showed a sharp increase in SB4 (42.14  µg/mL) compared to the control (20.19  µg/mL), reflecting treatment-specific metabolic responses, the reported differences were statistically significant at *p* < 0.05. These findings suggest that ultrasound-assisted fermentation promotes the release, transformation, and biosynthesis of key phenolic and organic acids, thereby enhancing the functional potential of sea buckthorn juice.

The present findings align well with the existing literature on sea buckthorn processing. El-Sohaimy, Shehata, Mathur, Darwish, Abd El-Aziz, Gauba and Upadhyay [24] demonstrated that fermentation with *Lactobacillus plantarum* promotes sugar liberation through microbial enzymatic hydrolysis of polysaccharides, resulting in quinic acid levels reaching approximately 19,800 µg/mL, gallic acid increasing to 64 µg/mL, ferulic acid rising to about 30 µg/mL, and catechin levels up to 31 µg/mL. These results are consistent with our observations of microbial-assisted saccharification enhancing phenolic and organic acid contents. However, the relationship between bioactive compounds and functional characteristics is complex. For instance, Yang, Yi, Wei, Lu, Yang, Yang, Zhao, Jiang and Tu [36] reported that phenolic compounds contributed minimally (less than 5 %) to sea buckthorn’s overall antioxidant capacity, which was predominantly driven by ascorbic acid present at roughly 400 µg/mL. Their study detected moderate levels of beneficial phenolics such as rutin (40 µg/mL), catechin (28 µg/mL) and chlorogenic acid (9 µg/mL), but collectively these accounted for a small fraction of the antioxidant activity. This suggests that while processing methods like ultrasound-assisted fermentation effectively increase specific phytochemicals as demonstrated in our study, the dominant antioxidant effects may still depend on non-phenolic components like ascorbic acid. Comprehensive profiling is essential to understand how processing affects functional properties. Combining ultrasound with specific probiotics (*L. plantarum, L. helveticus, L. paracasei*) during sea buckthorn fermentation significantly boosts phenolic compounds and organic acids. This enhances the berry's therapeutic and nutritional value, making it highly promising for novel functional foods and nutraceuticals delivering superior health benefits.

### 3.4 Quantification of organic acids in sea buckthorn juice using HPLC

**Fig. 5:**
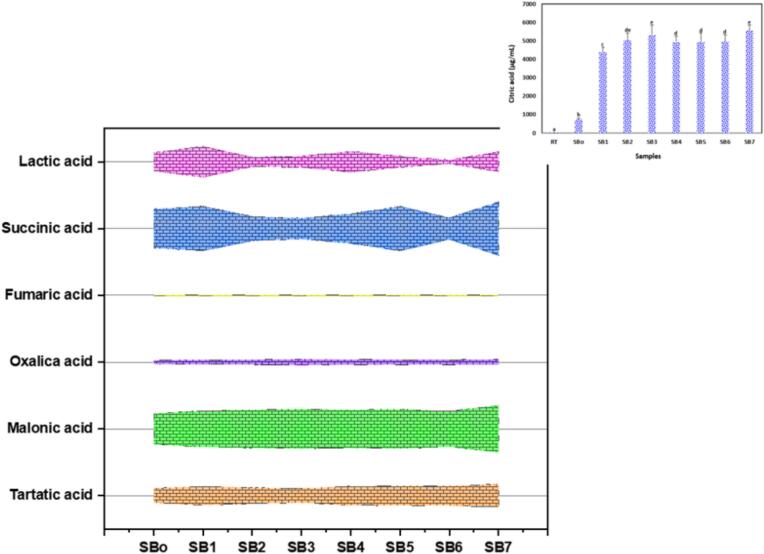
Organic acid profile of sea buckthorn juice samples (SBo–SB7) after ultrasound-assisted fermentation, showing variations in citric, lactic, succinic, oxalic, tartaric, malonic and fumaric acid concentrations due to microbial and ultrasonic processing. Means ± SD were represented, n = 3 replicates.

Organic acid profiling of sea buckthorn samples (SB_o_-SB_7_) revealed substantial treatment-induced variations, with citric acid predominating across all samples **(**Fig. 5**)**. Notably, citric acid exhibited a remarkable sevenfold increase, rising from 758.95 µg/mL in the unfermented control (SB_o_) to 5565.29 µg/mL in the ultrasound-assisted fermented sample SB_7_. Other organic acids displayed distinct trends: malonic acid increased from 108.00 to 165.07 µg/mL, tartaric acid rose from 51.58 to 81.79 µg/mL, and succinic acid fluctuated between 72.98 and 188.11 µg/mL, peaking in SB_7_. Oxalic acid reached its highest concentration in SB_3_ (20.40 µg/mL), while lactic acid initially increased in the ultrasound-assisted sample SB_1_ (108.11 µg/mL), then declined significantly to 12.62 µg/mL in SB_6_ before recovering to 69.83 µg/mL in SB_7_. Fumaric acid remained stable throughout the treatments, ranging from 3.84 to 4.00 µg/mL. A significance level of *p* < 0.05 confirmed the reliability of the noted alterations. These observed trends, especially the pronounced increases in samples SB_5_ through SB_7_, indicate that ultrasound-assisted fermentation combined with bacterial strains *L. plantarum*, *L. helveticus,* and *L. paracasei* effectively enhanced organic acid production, reflecting improved microbial metabolic activity and potentially augmenting the functional properties of sea buckthorn.

The observed increases in organic acid content align well with previous studies on fermented sea buckthorn. Tiitinen, Vahvaselkä, Hakala, Laakso and Kallio [37] demonstrated that fermentation with *L. plantarum* drives malolactic conversion, resulting in elevated lactic acid production (approximately 115 µg/mL) accompanied by corresponding decreases in malic acid. These changes are known to enhance both the metabolic profile and sensory characteristics of the product. Similarly, El-Sohaimy, Shehata, Mathur, Darwish, Abd El-Aziz, Gauba and Upadhyay [24] reported significant increases in organic acids during *L. plantarum* fermentation, with citric acid concentrations reaching nearly 5400 µg/mL and succinic acid around 165 µg/mL. They attributed these enhancements to intensified microbial enzymatic activity and fermentation metabolism. Collectively, these findings support our results, confirming that probiotic fermentation actively modulates the organic acid profile of sea buckthorns through targeted biochemical pathways. In contrast Wen, Huang, Li, Jiang, Shao, Zhang and Shan [35] observed that ultrasound-assisted extraction, while effective in increasing sugar and polysaccharide yields, did not substantially alter the organic acid composition of sea buckthorn. This discrepancy arises from their exclusive reliance on physical extraction without the involvement of microbial fermentation, which appears essential for driving the biochemical transformations responsible for modifying organic acid profiles. Thus, the integration of ultrasound with probiotic fermentation emerges as a critical factor in optimizing the functional and metabolic attributes of sea buckthorn.

### 3.5 Micromorphological properties of sea buckthorn

**Fig. 6:**
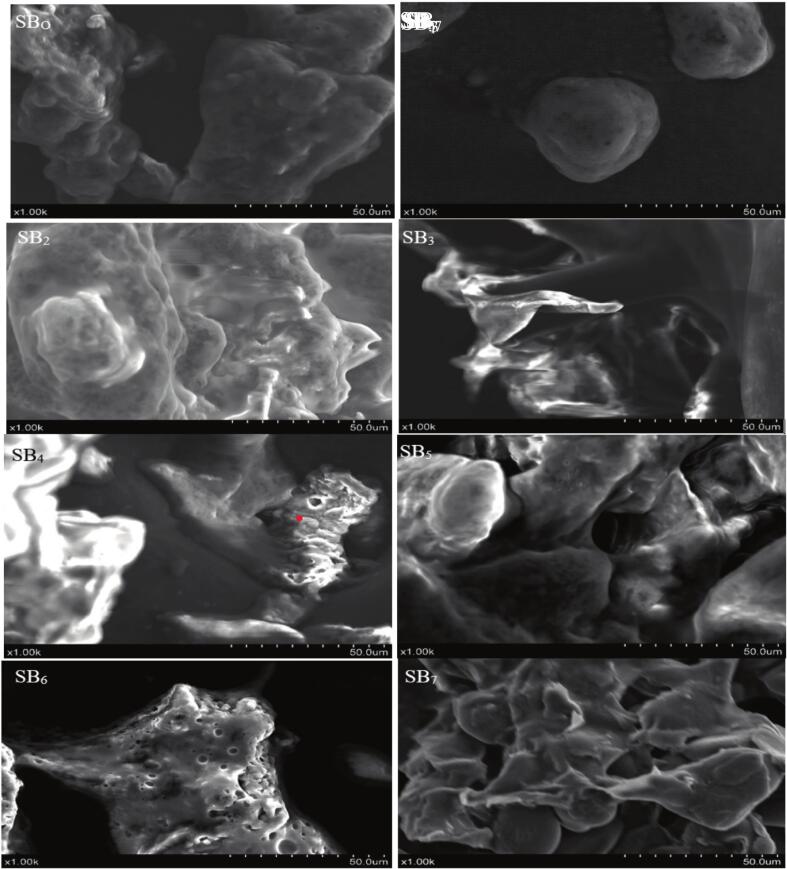
SEM images of sea buckthorn samples (SBo–SB7) showing structural changes after ultrasound-assisted fermentation, including trichomes, epidermal layers, seed coat porosity and pollen morphology, n = 1 replicate.

The scanning electron microscopy (SEM) examination shows the micromorphological features of varying sea buckthorn treatments (Fig. 6). SEM images of the ultrasound-treated sample and fermented SB_1_ and SB_2_ indicated uniform distribution of tightly packed trichomes over the berry surface. These trichomes were seen to be 50–70 µm in size and had a fine filamentous structure, supporting their role of creating a cover. These characteristics suggest a function in restricting water loss by transpiration and discouraging herbivorous activity by providing a physical barrier. Cross-section analysis of ultrasound-assisted fermented samples SB_3_ and SB_4_ presented a dense packing of epidermal cells under a distinctively visible cuticular wax layer, 2–3 µm in thickness. The waxy layer was smooth and continuous in morphology, which is usually associated with decreased permeability and enhanced drought tolerance and may indicate adaptive characteristics for water conservation in arid or semi-arid conditions. Ultrasound-assisted fermented samples SB_5_ and SB_6_ emphasized the architecture of the seed coat, presenting a rough, irregular surface with clear porosity. The heterogeneous texture of the seed coat, characterized by micro-sized pores and ridges, is assumed to be responsible for efficient nutrient retention. Ultrasound-assisted fermented sample SB_7_ illustrated the morphology of the pollen grain, typified by a reticulate exine pattern. The networked disposition of the exine ridges implies effective aerodynamic properties, which are vital in successful pollination, especially in windy environmental conditions.

The present outcomes are consistent with the previous study of Zeb and Malook [38], as indicated by the dark brown, glossy seed coat of sea buckthorn seeds, which measured ∼ 4.3 mm in length and 2.4 mm in breadth, as observed through SEM analysis. The study offers insightful details on sea buckthorn seed morphology, supporting findings of porosity and structural elements that facilitate oil storage. Epicuticular and intracuticular components make up the wax layer, which helps plants retain water and protect against environmental stressors, according to research by Bellucci, Nanni, Bitocchi, Rossi and Papa [39] on plant cuticular waxes. These protective roles are consistent with the smooth, continuous wax layer seen in sea buckthorn samples. Another investigation of Gama-Arachchige, Baskin, Geneve and Baskin [40] determined that numerous exine features, such as reticulate structures, which improve aerodynamic efficiency for wind pollination, have been reported by studies on pollen morphology. These results are in accordance with the present findings, reticulate exine pattern found in sea buckthorn pollen grains, highlighting their adaptation to anemophilous pollination techniques. To sum up, SEM analysis demonstrated distinct micromorphological variations in Sea Buckthorn involving the existence of filamentous trichomes, porous seed coat structures, compact epidermal cells beneath a well-defined cuticular wax layer and reticulate exine patterns on pollen grains. These characteristics suggest improved functional roles in nutrient retention, water conservation, and reproductive efficiency. The observed changes using *L. plantarum*, *L. helveticus,* and *L. paracasei*, which have been stated to affect the surface morphology and structural integrity across microbial activity and associated biochemical alterations during fermentation.

### 3.6 FTIR of sea buckthorn

**Fig. 7:**
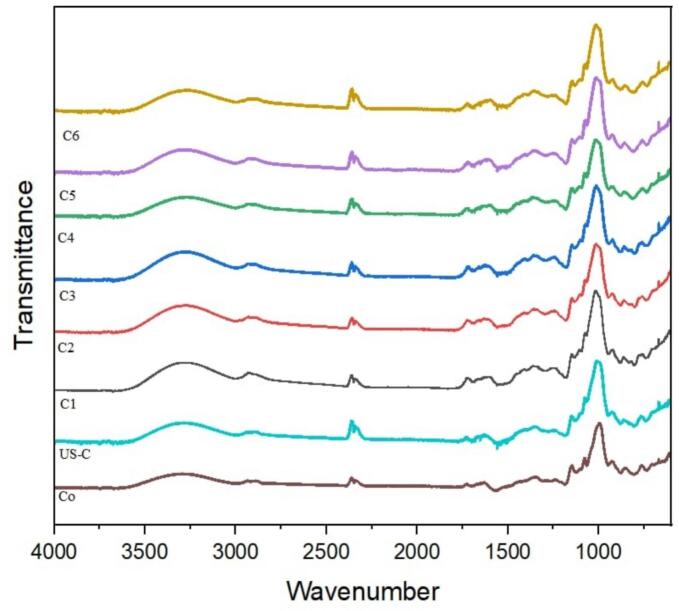
FTIR spectra of sea buckthorn samples (SBo–SB7) illustrating functional group shifts associated with ultrasound-assisted fermentation, reflecting structural changes in polysaccharides, proteins and ester linkages, n = 1 replicate.

The FTIR analysis of sea buckthorn treatments (unfermented control SB_o_, ultrasound-assisted and fermented samples SB_1_-SB_6_) revealed distinct spectral differences, signifying compositional and structural changes induced by various samples **(**Fig. 7**)**. All the treatments indicated a broad absorption band around 3300–3400 cm^−1^, corresponding to O–H stretching vibrations, indicative of hydroxyl-containing components such as polysaccharides and phenolics.

Peaks near 2920 cm^−1^ and 2850 cm^−1^ were ascribed C–H stretching of methyl and methylene groups, usually correlated with lipids and fatty acid chains. Distinctively in SB_5_ and SB_6_ exhibited high absorption around 1740 cm^−1^, expressing C=O stretching of esters or carboxylic acids, which may be linked to improved release or structural exposure of pectin and other esterified components. Moreover, a band near 1640 cm^−1^ was visible among all treatments, related to amide I or C=C stretching, reflecting the existence of proteins or conjugated systems. The substantial differences were noted in the fingerprint region between 1000–1200 cm^−1^, where SB_5_ and SB_6_ showed the intense transmittance peaks. These peaks are lined with C–O–C and C–O stretching vibrations, indicative of increased carbohydrate and polysaccharide content or structural accessibility. Across these samples, the SB_6_ presented the high spectral intensity, particularly in the fingerprint region, revealing the significant structural breakdown and exposure of bioactive compounds, resulting from enhanced consequences of processing.

The present investigation is in agreement with the work of Wang, Cheng, Wu, Fang, Rahman, Hao and Zhang [41], who noted the similar FTIR characteristics in sea buckthorn polysaccharides, involving the strong O–H, C–H and C–O–C absorption bands indicative of their complex polysaccharide matrix. Another study of Kholodov, Kholodova, Gorlov, Shakhbazova, Mosolova, Glushenko and Mosolova [42], reported that ester-related C=O bands around 1740 cm^−1^ and pronounced signals in the 1000–1200 cm^−1^ region in Sea Buckthorn pectin, affirming its substantial galacturonic acid presence and semi-crystalline pattern. In opposition the study conducted by Luo, Zhang, Xie and Chen [43], observed that under controlled extraction and fermentation conditions, the FTIR spectra of Sea Buckthorn polysaccharides demonstrated reduced peak intensities and broader profiles, particularly in the fingerprint and hydroxyl regions, showing a disruption of structural order maybe due to excessive processing. To wrap up, the FTIR graph revealed significant compositional and structural alterations in Sea Buckthorn, characterized by improved absorption intensities, primarily in regions associated with carbonyl, hydroxyl, and polysaccharide-related functional groups. These modifications, especially the pronounced spectral shifts in the fingerprint region, reflect an increased exposure of bioactive components and breakdown of integrated structures. Utilizing the *L. plantarum*, *L. helveticus* and *L. paracasei*, which have exhibited altered molecular structures and enhanced the accessibility of functional compounds through enzymatic and microbial activity during fermentation.

**Fig. 8:**
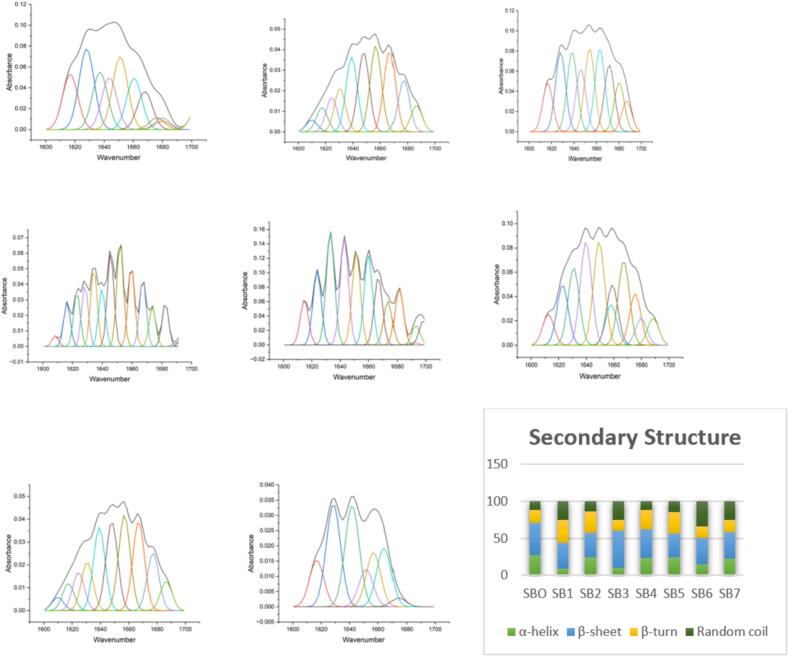
Secondary structure distribution of sea buckthorn samples (SBo–SB7) showing processing-induced variations in α-helix, β-sheet, β-turn and random coil content, n = 1 replicate.

Secondary structure analysis of sea buckthorn samples (SB_o_–SB_7_) revealed notable conformational changes under different processing conditions (Fig. 8). The unfermented control (SB_o_) exhibited the most ordered protein structure, with high α-helix (27.93 %) and β-sheet (43.51 %) content and minimal random coil (11.67 %), indicating a preserved native conformation. In contrast, SB_1_ and SB_3_ showed the most pronounced structural disruption, with α-helix content reduced to 9.14 % and 9.87 % and random coil content increased to 24.70 % and 25.57 %, respectively, suggesting significant protein unfolding. SB_3_ also exhibited the highest β-sheet content (50.36 %), potentially reflecting stress-induced aggregation. SB_6_ displayed the most disordered structure, with peak random coil (33.94 %) and reduced α-helix (14.49 %). Samples SB_2_, SB_4_, SB_5_ and SB_7_ maintained stable conformations, with α-helix levels between 22.34–24.97 % and moderate random coil content (11.92–24.77 %), indicating partial structural retention. Notably, β-turn content varied, with SB1 showing the highest (31.05 %), as a compensatory folding mechanism, and SB_3_ the lowest (14.19 %), aligning with its unfolded state. Overall, treatments SB_1_, SB_3_ and SB_6_ underwent the most severe conformational changes, while the remaining samples preserved intermediate secondary structural stability.

These structural findings are well supported by previous FTIR-based investigations on protein secondary structure. Yu, McKinnon, Soita, Christensen and Christensen [44] reported similar conformational changes in flaxseed proteins using synchrotron-FTIR, where thermal roasting led to a notable reduction in α-helix content from 47.1 % to 36.1 %, accompanied by an increase in β-sheet structures from 37.2 % to 49.8 %. Likewise, Wang, Liu, Ren, Zhang, Stephanie, ZHANG and GUO [45] observed progressive proteolytic changes in cheddar cheese during ripening, with α-helix content decreasing from 32.4 % to 24.6 % and β-sheet content increasing from 35.8 % to 43.1 %, reflecting structural reorganization. In contrast, reported remarkable structural stability in flaxseed proteins during thermal processing, with only minor shifts in α-helix (35.9 % to 34.7 %) and β-sheet (38.2 % to 39.0 %), suggesting high resilience to processing-induced denaturation. This divergence highlights the importance of protein source and inherent molecular properties in dictating structural adaptability. In the present study, the application of multi-strain fermentation involving *L. plantarum, L. helveticus,* and *L. paracasei* induced marked modifications in protein conformation, characterized by a decrease in α-helix content and a corresponding rise in β-turn and random coil structures. These transitions reflect varying degrees of protein unfolding and increased molecular flexibility, attributed to microbial enzymatic activity and cavitation effects of ultrasound. The observed variability across treatments further highlights the dynamic influence of processing strategies on protein structural behavior in sea buckthorn juice. Such structural transformations may directly influence the techno-functional and bioactive properties of sea buckthorn juice, offering opportunities for the development of value-added functional beverages.

### 3.7 XRD analysis of sea buckthorn

**Fig. 9:**
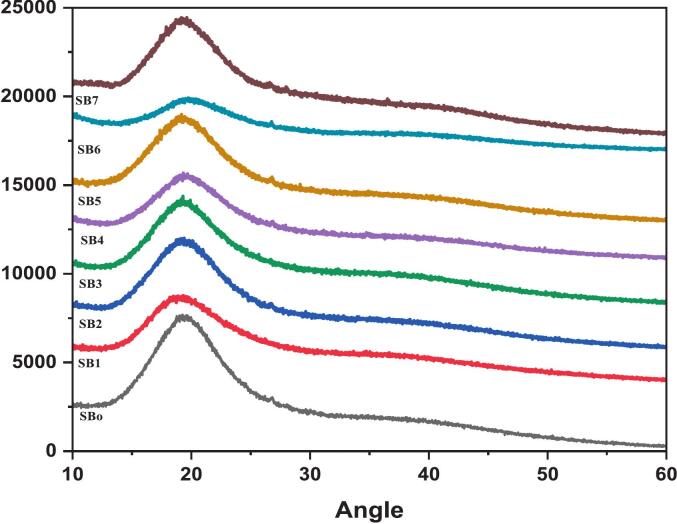
XRD patterns of sea buckthorn samples (SBo-SB7), illustrating treatment-induced changes in crystallinity, with SB7 exhibiting the highest peak intensity, n = 1 replicate.

The X-ray diffraction (XRD) investigation of sea buckthorn treatments (SB_o_-SB_7_) revealed significant changes in their crystalline structure **(**Fig. 9**)**. All the samples illustrated in a graph revealed a broad diffraction peak in the 2θ range of ∼ 20-22°, suggesting the presence of amorphous and semi-crystalline constituents, attributed to natural polysaccharides and cell wall components. The unfermented control sample SB_o_ exhibited a low peak intensity, reflecting an amorphous structure. Conversely, the ultrasound-assisted fermented sample SB_7_ indicated progressively high diffraction intensities, displaying the prominent peak, subsequently accompanied by SB_6_ and SB_5_. This implies that the processing techniques employed were responsible to a great degree for molecular restructuring or semi-crystalline structure. The increased peak sharpness and intensity in these treatments reveal improved internal structural configuration, which may be due to the combined effects of fermentation and ultrasound treatments.

The present findings are coherent with the investigation of Zhu, Ji, Yuen, Yuen, Yuen, Wang, Smith and Peng [46], who elucidates the that sea buckthorn insoluble dietary fiber treated with ball milling and cellulase indicated the increased crystallinity, as reflected by XRD analysis, demonstrating improved structural arrangement. Likewise, another study Wang, Cheng, Wu, Fang, Rahman, Hao and Zhang [41], noted that ultrasound-assisted extraction led to changes in the structural characteristics of sea buckthorn polysaccharides, contributing to enhanced functional properties. In contrast to the present outcomes, the study by Luo, Zhang, Xie and Chen [43] have shown divergent results, who found that certain samples resulted in a reduction in crystallinity of sea buckthorn polysaccharides, as identified by XRD graph, implying the impact of processing techniques on structural features can vary depending on certain conditions and treatment parameters. To recapitulate, the present study's findings regarding increased crystallinity in ultrasound-assisted fermented treatments of sea buckthorn are consistent with some prior investigations due to the co-culture bacteria that are *L. plantarum, L. helveticus,* and *L. paracasei*, which have been observed to influence the microstructural integrity of plant matrices through enzymatic and biochemical interactions during fermentation, contributing to improved functional and morphological traits.

### 3.8 Molecular docking

**Fig. 10:**
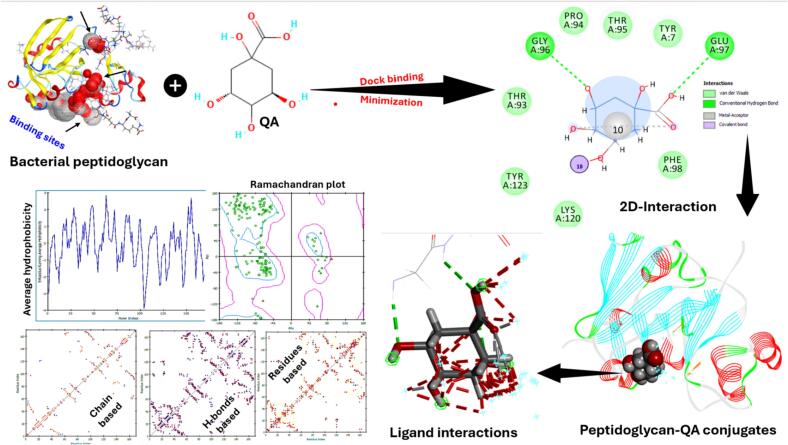
Molecular docking of the key polyphenols in sea buckthorn with the bacterial peptidoglycan.

Molecular docking was done to theoretically investigate the interaction between bacterial proteins and polyphenols of sea buckthorn juice. Fig. 10 comprehensively illustrates the molecular interplay between peptidoglycan and QA, employing multiple analytical techniques to dissect binding dynamics and affinity. The workflow incorporates critical validation steps, including Ramachandran plot analysis to assess protein structural quality, ensuring that most amino acid residues fall within energetically favorable conformational regions. The binding site analysis reveals specific amino acid interactions, including THR A:93–95, TYR A:7–123, Lys A120, PHE A:98, GLY A:96, GLU A:97, and Pro A:94, indicating key residues involved in ligand recognition. Meanwhile, Gly96 and Glu97 interacted with QA via H-bonds.

The 2D-interaction diagram provides a detailed visualization of intermolecular forces, including H-bonds, hydrophobic interactions, and van der Waals contacts, which are essential for understanding binding affinity and selectivity. The presence of multiple interaction types (depicted in green for conventional H-bonds, pink for weak interactions, and other color-coded representations) suggests a stable protein–ligand complex formation. The observed binding score of this conjugate was −4.55, coupled with an energy affinity of −2.09 kcal/mol, which points to a moderately stable interaction, suggesting functional implications for the resulting conjugates.

### 3.9 Electronic-nose (E-nose) analysis of sea buckthorn

**Fig. 11:**
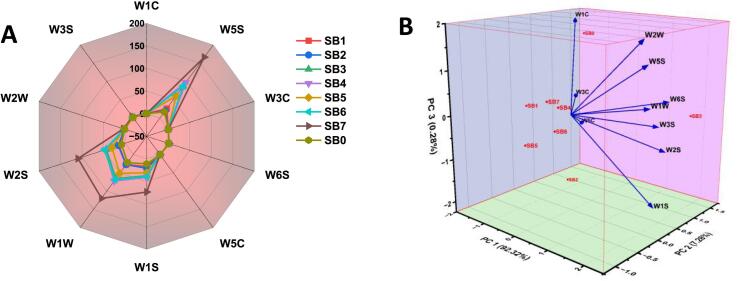
(A) Radar plot showing electronic nose sensor responses to volatile compounds in sea buckthorn samples (SBo–SB7). (B) Principal Component Analysis (PCA) of E-nose data illustrating clear differentiation in volatile profiles among treatments.

As illustrated in the radar diagram **(**Fig. 11**A)**, the evaluation of the sea buckthorn treatments (SB_1_–SB_7_) and the unfermented control sample (SB_o_) using the E-nose displayed distinct volatile profiles **(**Fig. 11**B)**. The SB_7_ ultrasound-assisted fermented sample showed high sensor responses across all treatments, especially with W5S, which is vulnerable to nitrogen oxide, and W2S, which identifies ethanol and aromatics. With just slight variations between the various sensors, samples SB_1_-SB_6_ showed moderate and consistent sensor readings. However, SB_o_ exhibited low sensor activity, remaining near 0 for every sensor reading. Particularly, sensors W1C, W3S, and W5S are effective in distinguishing the aroma characteristics of the sea buckthorn treatments, signifying differences in production trends of the volatile organic compound (VOC) across the samples. According to the radar graph, the sea buckthorn treatments had a varied spectrum of volatiles.

The present findings are congruent with the research of Zhou, Zhang, Liu, Ji, Zhang and Yang [47], who elucidate the E-nose odor profiles of sea buckthorn pulp that was dried using various methods. The results showed that different drying methods affected the volatile compounds in the pulp, with vacuum-freeze drying maintaining the desired best aroma qualities. The potential of the E-nose is to identify minimal changes in volatile profiles was highlighted by the PCA of the E-nose, which exhibited distinct sample classes based on drying. The present outcomes are coherent with the latest reports by Murtaza, Yaqoob, Mubeen, Sameen, Murtaza, Rehman, Alsulami, Korma, Khalifa and Ma [21], who utilized *L. halvaticus* as a co-culture bacterium and produced a significant amount of volatile compounds after 48 h in both fermented and ultrasound-treated samples. The E-nose analysis revealed distinct differences in the volatile profiles of sea buckthorn treatments, with notable enhancement in aroma-related sensor responses compared to the unfermented control sample. The increased activity, particularly in sensors responsive to ethanol, nitrogen oxides and aromatics, suggests more complex and diverse volatiles.

### 3.10 Color assessment of sea buckthorn

**Fig. 12:**
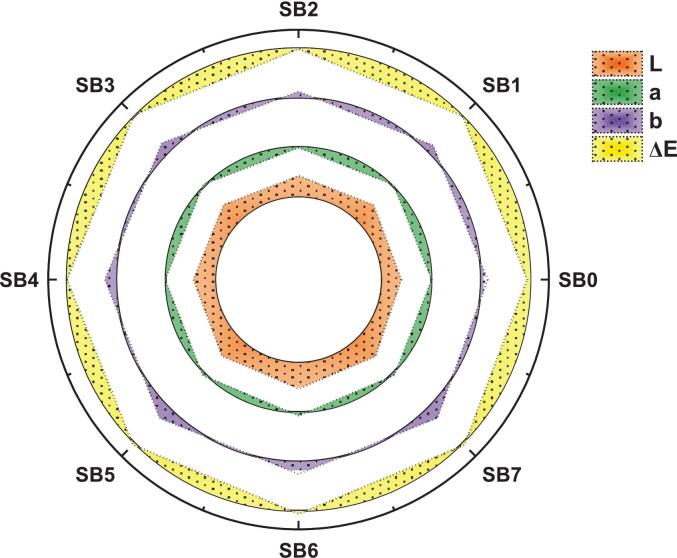
Colorimetric parameters (L*, a*, b* and ΔE) of sea buckthorn samples (SBo–SB7), highlighting changes in total color difference (ΔE) due to fermentation and ultrasound treatment, n = 3 replicates.

The color parameters L, a*, b*, and ΔE of sea buckthorn samples (SB_o_ to SB_7_) were systematically evaluated **(**Fig. 12**)**. A gradual increase in lightness (L) was observed, rising from the unfermented control sample SB_o_ (46.75 ± 0.94) to the ultrasound-assisted fermented sample SB_6_ (58.59 ± 1.17). This indicates that more intensive processing treatments led to a noticeably brighter appearance. The a* values, reflecting the red-green axis, increased significantly from SB_2_ (11.16 ± 0.22) to SB_6_ (18.77 ± 0.38), suggesting a shift toward a deeper red hue. Concurrently, the b* values, indicative of the yellow-blue axis, rose from SB_2_ (23.49 ± 0.47) to SB_5_ (37.44 ± 0.75), enhancing overall color saturation. The highest ΔE values were observed in SB_6_ (17.66 ± 0.35), SB_7_ (16.79 ± 0.34), and SB_5_ (16.76 ± 0.34), indicating a marked deviation from the control (SB_o_). All observed differences were statistically meaningful (*p* < 0.05). These pronounced changes in chromaticity are attributed to the effects of fermentation and ultrasonic processing. Overall, the findings demonstrate that the applied processing methods significantly influence the color profile of sea buckthorn, enhancing its reddish-yellow intensity and visual brightness.

The present results are in opposition to the earlier findings of Máté, Selimaj, Simon, Szalóki-Dorkó and Ficzek [48], who evaluated the color of sea buckthorn berries of different types. The cultivar 'Clara' demonstrated a vivid orange color with high L (57.03 ± 0.04), a* (14.69 ± 1.47), and b* (53.24 ± 1.66). On the other hand, 'Ascola' exhibited lower values for all these metrics. The modifications were attributed to deviations in the cultivars' carotenoid content. Another finding is somewhat in line with Zhang, Chen, Chen, Gao, Cheng and Qu [49], who revealed that the color characteristics of sea buckthorn juices from various geographical regions were examined. Fermented Sea Buckthorn juice showed significantly higher L, a, b* values and ΔE, indicating a brighter, more vibrant color than the control. This improvement, linked to changes in anthocyanin and carotenoid concentrations, aligns with studies showing specific co-cultures reduce browning and enhance pigment stability for better visual appeal.

### 3.11 Organoleptic assessment of sea buckthorn

**Fig. 13:**
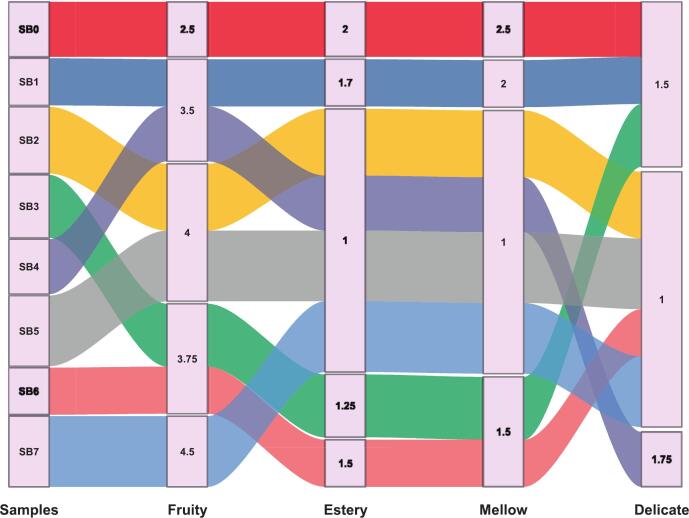
Sensory profiling of aroma attributes in sea buckthorn samples (SBo–SB7), showing enhanced perception of fruity and floral notes following fermentation treatments.

The sensory characteristics of sea buckthorn samples (SB_o_-SB_7_) showed significant differences among five aroma properties that are mellow, estery, fruity, delicate, and floral Fig. 13. Across these parameters, the fruity trait was improved in ultrasound assisted and fermented treatments, specifically in SB_1_, SB_3_ and SB_5_, that was reported high intensity scores around 4.5, as compared to unfermented control sample (SB_o_), which had a notably low fruity score of 2.5. This indicates that processing techniques like fermentation or ultrasound-assisted sample preparation aided in the synthesis or release of fruity volatile constituents, which enhances the sensory appeal. However, estery notes, usually linked with fruity, sweet aromas, remained restrained among all treatments, with values typically ranging between 1.0 and 2.0, exhibiting only a slight increase in SB_1_ and SB_2_. The mellow characteristic, which shows smooth and rounded aromatics, was also generally low across the treatments, though SB_o_ demonstrated moderately higher values than the treated samples, implying that the treatments may have shifted the odor profile toward more dominant or vibrant notes at a loss of mellow qualities. Likewise, the delicate aromas, those subtle and light volatiles, were minimally perceived, with all samples scoring around 1.0 to 2.0. SB_6_ showed a negligible increase; this could be owing to processing effects that maintain aromatics. The floral traits were improved specifically in SB_3_, SB_5_, SB_6_ and SB_7_ with scoring range of 4.0 and 4.5 in contrast to the value of SB_o_ and SB_1_; that is 3.0, suggesting that the treatments were particularly effective in elevating the presence of floral volatiles, derived from the phenylpropanoid pathways or fermented by-products. Significant changes were observed among the treatments, as indicated by *p* < 0.05.

The current study is in aversion to the findings of Laaksonen, Knaapila, Niva, Deegan and Sandell [50], who identified over 50 different compounds in sea buckthorn, with esters and aldehydes giving it a significant part of its aroma. There is also a floral note, which is less strong but fits with the berry's high terpene content. The changes in fruity and smooth qualities across different samples could be due to processing differences. Our present results are supported by Liu, Zhu and Zhu [51], who investigated the impact of ultrasound-pretreated fermentation on hawthorn pulp by using LAB bacteria and observed that the ultrasonicated-fermented hawthorn pulp had a higher overall acceptability and a positive influence on sensory parameters of the ultrasound-fermented samples. The sensory profiling results are in accordance with Murtaza, Yaqoob, Mubeen, Sameen, Murtaza, Rehman, Alsulami, Korma, Khalifa and Ma [21] after fermentation and triple-frequency ultrasound treatment assisted using co-culture bacteria (*L. helveticus* and *L. plantarum*), which produce volatile flavor compounds, through these mechanisms are considered to enhance overall sensory appeal. Overall, the findings showed that certain treatments improved the fruity and floral profiles of sea buckthorns, particularly those involving *L. plantarum*, *L. helveticus,* and *L. paracasei* significantly enhances the flavors, sensory quality, and consumer perspective, whereas the delicate, mellow and estery qualities continues less dominant. These co-culture bacteria are known to catalyze the production of flavor-improving volatile compounds aligning well with findings from previous studies that reported similar improvements in aroma notes through fermentation. The sensory profiling also exhibited favorable scores in terms of aroma, taste, and overall acceptability, showing potential consumer acceptance without compromising organoleptic quality. However, large-scale validation, shelf-life analysis, and compliance with microbial safety and labeling regulations are essential next steps. Addressing these aspects will ensure the transition of this thriving technology from laboratory scale to commercial reality, encouraging the creation of value-added, probiotic-enriched juice items that meet both nutritional and regulatory criteria.

### 3.12 Limitations and future perspectives for industrial application

Although multi-frequency ultrasound offers notable processing benefits, cavitation may induce free radical formation. This study employed pulsed sonication, moderate temperatures, and limited exposure times to mitigate this risk; however, further work is needed to assess ROS generation and oxidative stability in treated products [13]. While the process improved the functional and sensory quality of sea buckthorn juice, industrial application presents challenges. Microbial stability over time has not been evaluated, despite the use of GRAS strains *(L. plantarum, L. helveticus, L. paracasei*), warranting microbial profiling during storage. Shelf-life studies are also essential, as ultrasound and fermentation could influence product stability. Scalability remains a constraint, requiring optimization of energy use, equipment design, and processing time. Economic viability should be supported by cost-benefit analyses, including equipment amortization and operational efficiency. Consumer acceptance beyond sensory evaluation, especially for clean-label functional products, must be explored. Finally, although ultrasound is considered green, its cumulative energy impact with fermentation warrants assessment via life cycle analyses. Addressing these factors is key to enabling industrial translation of this promising bioprocess.

### 3.13 Conclusion

This study demonstrates that multi-frequency ultrasound significantly enhances the fermentation efficiency and bioactive compound content of sea buckthorn juice, while improving sensory and textural properties. Microstructural analysis and computational modeling provided mechanistic insights into observed changes. These findings lay the groundwork for future development of functional fermented beverages using non-thermal technologies.

## Ethics statement

No animal and human experiments. The sensory study was performed in a safe and hygienic food production environment, with the generous assistance of the Laboratory of Food Nutrition and Clinical Research, Institute of Seafood, Zhejiang Gongshang University, Hangzhou, China.

## CRediT authorship contribution statement

**Sanabil Yaqoob:** Writing – review & editing, Writing – original draft, Methodology, Investigation, Formal analysis, Conceptualization. **Aysha Imtiaz:** Writing – review & editing, Methodology, Investigation, Data curation, Conceptualization. **Kanza Aziz Awan:** Writing – original draft, Visualization, Validation. **Hiba Naveed:** Writing – review & editing, Writing – original draft, Methodology, Investigation. **Ahmad Faraz:** Writing – review & editing, Visualization, Validation, Software. **Remah Sobhy:** Writing – review & editing, Writing – original draft, Visualization, Validation, Project administration, Funding acquisition. **Jian Ya Qian:** Writing – review & editing, Writing – original draft, Supervision, Resources, Project administration, Funding acquisition. **Akmal Nazir:** Writing – review & editing, Writing – original draft, Visualization, Validation, Supervision, Software, Resources, Project administration. **Qing Shen:** Writing – review & editing, Writing – original draft, Visualization, Validation, Supervision, Software, Resources, Project administration, Funding acquisition.

## Declaration of competing interest

The authors declare that they have no known competing commercial interests or personal relationships that could have appeared to influence the work reported in this paper.
